# The Role and Clinical Relevance of Osteopontin in Allergic Airway Diseases

**DOI:** 10.3390/jcm12062433

**Published:** 2023-03-22

**Authors:** Yang Liu, Li Fu, Zheng Liu

**Affiliations:** 1 Department of Otolaryngology-Head and Neck Surgery, Tongji Hospital, Tongji Medical College, Huazhong University of Science and Technology, Wuhan 430030, China; 2 Institute of Allergy and Clinical Immunology, Tongji Hospital, Tongji Medical College, Huazhong University of Science and Technology, Wuhan 430030, China; 3 Hubei Clinical Research Center for Nasal Inflammatory Diseases, Wuhan 430030, China

**Keywords:** osteopontin, inflammation, biomarker, therapeutic target, pathogenesis

## Abstract

The airway epithelium is exposed to numerous external irritants including infectious agents, environmental allergens, and atmospheric pollutants, releasing epithelial cytokines including thymic stromal lymphopoietin (TSLP), IL-33, and IL-25 and initiating downstream type 2 (IL-4, IL-13, and IL-5) and IgE-driven pathways. These pathways trigger the initiation and progression of allergic airway diseases, including chronic rhinosinusitis with nasal polyps (CRSwNP), allergic rhinitis (AR), and allergic asthma. However, the use of biological agents that target downstream cytokines, such as IL-5, IL-4, and IL-13 receptors and IgE, might not be sufficient to manage some patients successfully. Instead of blocking downstream cytokines, targeting upstream epithelial cytokines has been proposed to address the complex immunologic networks associated with allergic airway diseases. Osteopontin (OPN), an extracellular matrix glyco-phosphoprotein, is a key mediator involved in Th1-related diseases, including systemic lupus erythematosus, multiple sclerosis, inflammatory bowel disease, and rheumatoid arthritis. Emerging evidence, including ours, indicates that epithelial-cell-derived OPN also plays an essential role in Th2-skewed airway diseases, including CRSwNP, AR, and allergic asthma involving the Th17 response. Therefore, we reviewed the current knowledge of epithelial-cell-derived OPN in the pathogenesis of three type-2-biased airway diseases and provided a direction for its future investigation and clinical relevance.

## 1. Introduction

Clinically, type-2-related airway diseases manifest as chronic rhinosinusitis with nasal polyps (CRSwNP), allergic rhinitis (AR), and allergic asthma [1,2]. The etiology of these disorders remains ill-defined and is influenced by multiple factors, including genetic susceptibility, viruses, allergens, infection, and others [3,4]. CRSwNP is a common clinical entity of the upper airway and is characterized by the infiltration of polyps by numerous eosinophils [5]. AR and allergic asthma are caused by various inflammatory networks related to IgE-mediated activity in response to allergens in the upper and lower airways, respectively [6,7]. These three allergic airway diseases display Th2 cytokine responses and involve a range of immune cells and cytokines [8]. Notably, allergic airway diseases have been recognized as Th2/Th17 mixed diseases [9]. 

In response to endogenous and extrinsic airway irritants, epithelial cells are activated and produce cytokines which, in turn, might induce downstream Th2- and IgE-related responses [10]. The upstream epithelial cytokines, such as thymic stromal lymphopoietin (TSLP), IL-33, and IL-25, are known to play an essential role in this process [11]. Recently, various biologics have targeted the Th2 pathway and IgE, including omalizumab, mepolizumab, benralizumab, and dupilumab [12,13], and have demonstrated significant promise for treating patients with allergic airway diseases [14,15]. However, despite these promising advances, approximately 30% of patients with severe asthma who receive biologics do not exhibit significant improvement in their acute exacerbation rate, and only 50% of patients with CRSwNP respond to these biologics [16,17]. The limitations of these biologics might be due to blockage of the downstream immune cascade pathway, leaving other pathways still active, and because the partial inhibition of the type 2 pathway might not be sufficient to manage all patients [17]. Biological therapies targeting upstream cytokines such as TSLP, IL-33, and IL-25 have greatly improved the therapeutic results for patients with allergic airway diseases in clinical trials [18,19,20]. One clinical study demonstrated that AMG157, a humanized anti-TSLP monoclonal immunoglobulin G2k, reduced bronchospasms and bronchial pathology in individuals with mild allergies [20]. Therefore, an enhanced understanding of the cytokines expressed by a dysfunctional respiratory epithelium could facilitate the development of novel clinical therapies.

OPN, an extracellular matrix phosphor-glycoprotein, has long been recognized as a key mediator in Th1-related immunity and is involved in Th1-related diseases, including systemic lupus erythematosus (SLE), multiple sclerosis (MS), inflammatory bowel disease (IBD), and rheumatoid arthritis (RA) [21,22,23,24,25]. Findings suggest that OPN might be a potential biomarker for monitoring the severity of SLE [25]. In RA patients, plasma OPN levels correlate with bone inflammation and serve as a biomarker for disease severity [22]. In animal models of RA, targeting OPN with small interfering RNA (siRNA) or neutralizing antibodies to the relevant epitopes resulted in a significant inhibition of joint inflammation [26,27]. Recent works, including ours, demonstrated that epithelial-cell-derived osteopontin (OPN) plays a critical role in CRSwNP, AR, and allergic asthma [28,29,30,31,32].

In this review, we summarized the current knowledge about the role of epithelial-cell-derived OPN during the development of the airway mucosal type 2 immune response. We also discussed the clinical potential of modulating OPN to treat CRSwNP, AR, and allergic asthma.

## 2. OPN Gene & Structure

The OPN gene, also called SPP1, is located on human chromosome 4q13 and mouse chromosome 5 and has seven exons, with exon 1 being noncoding [33,34,35]. In humans, SPP1 yields five OPN isoforms due to alternative splicing: OPN-a (full length); OPN-b (lack of exon 5); OPN-c (lack of exon 4); OPN-4 (lack of exons 4 and 5); and OPN-5 (with an extra exon located between canonical exons 3 and 4) [36]. All the isoforms have been studied in the cancer field and confirmed to have varied expression and function in different cancer types; only OPN-a has been investigated in non-tumor diseases [37,38]. Its molecular weight ranges from 44 to 75 kDa, depending on alternative splicing and post-translational modifications [39].

## 3. Forms of OPN

OPN is expressed by immune cells including B cells, T cells, natural killer (NK) cells, epithelial cells, and fibroblasts [40]. Two forms of OPN have been identified [41]. Secreted OPN (sOPN) is generated by the translation of the full-length SPP1 mRNA, whereas intracellular OPN (iOPN) is produced by translation downstream of the non-AUG codon [42]. The two forms exhibit different expressions in various cell types: dendritic cells (DCs) have a high expression of iOPN but low levels of sOPN, whereas the reverse is true for activated T cells [43]. sOPN exerts its biological effects through several receptors, while iOPN primarily acts as an adaptor or scaffolding protein involved in cytoskeletal rearrangement and signal transduction pathways downstream of innate immune receptors, such as Toll-like receptors (TLRs) [44,45]. 

## 4. OPN Receptors

**Figure 1:**
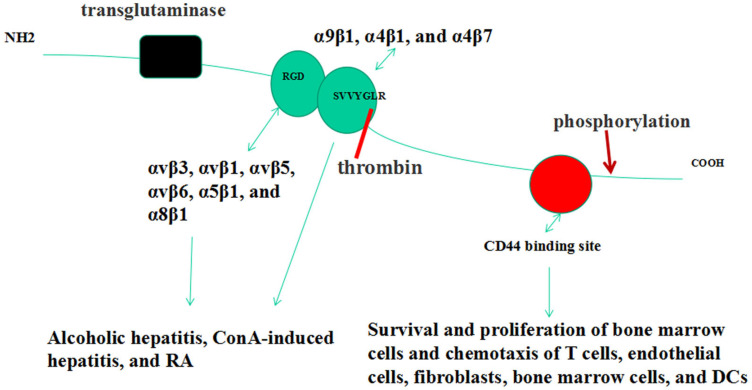
A schematic representation of osteopontin structure and thrombin cleavage site. OPN interacts with two groups of receptors, integrins (in green) and CD44 (in red), to exert its biological function. Thrombin cleavage of OPN exposes the SVVYGLR sequence. OPN might undergo posttranslational modifications, including phosphorylation, polymerizations by transglutaminase (in black), and cleavage (thrombin). RA: rheumatoid arthritis; DCs: dendritic cells.

OPN has two critical integrin binding sequences, including arginine-glycine-aspartic acid (RGD) and serine-valine-valine-tyrosine-glutamate-leucine-arginine (SLAYGLR in mice, SVVYGLR in humans) (Figure 1) [46,47]. Thrombin cleavage of OPN exposes the latter integrin binding sequence [48]. OPN interacts with integrin receptors, includingαvβ3, αvβ1, αvβ5, αvβ6, α5β1, and α8β1, via the RGD motif and interacts with α9β1, α4β1, and α4β7 receptors via SVVYGLR (SLAYGLR in mice) [46,47,48]. These interactions have been implicated in inflammatory disorders such as ConA-induced hepatitis and MS [49,50]. CD44 isoforms act as another critical receptor group for OPN [51]. The interaction between CD44 and OPN regulates IL-10 production in T cells, as demonstrated in an experimental autoimmune encephalomyelitis model [52]. This interaction enhances the survival and proliferation of bone marrow cells and is required for the chemotaxis of T cells, endothelial cells, fibroblasts, bone marrow cells, and DCs [53,54,55,56,57,58].

## 5. OPN Post-Translational Modifications

Phosphorylations and O-glycosylations are the primary post-translational modifications (PTMs) for OPN (Figure 1) [59]. Phosphorylation might influence the interaction between OPN and its receptors, bone mineralization, and tumor metastasis [60,61]. O-glycosylations modulate numerous cellular functions, such as binding interactions [62]. Another important post-translational modification for OPN is polymerization. OPN increases its adhesive properties by generating polymers crosslinked by tissue transglutaminase-2 [63]. By binding integrin α9β1, OPN polymers contribute to neutrophil recruitment [64]. Whether this polymerization affects other immune responses remains unexplored.

## 6. Functions of OPN

**Figure 2:**
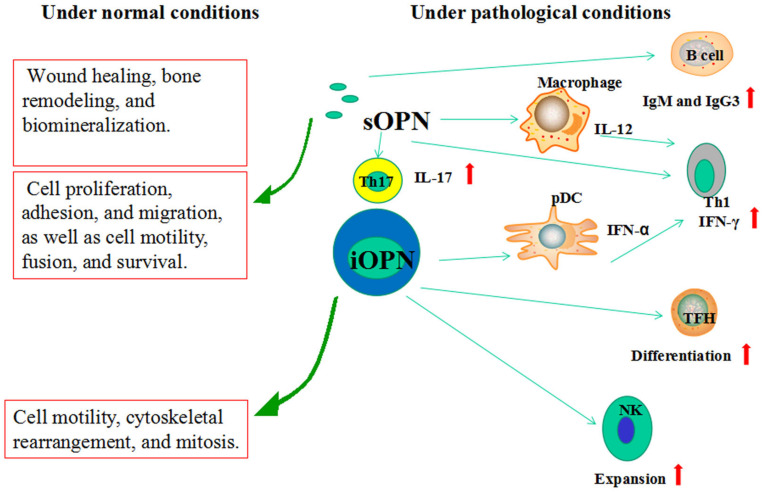
Effect of the two forms of OPN. sOPN and iOPN trigger a functional response under normal conditions and pathological conditions. OPN: osteopontin; sOPN: secreted osteopontin; iOPN: intracellualr osteopotin; pDC: plasmacytoid dendritic cell; cDC: conventional dendritic cell; TFH:T follicular helper cell.

OPN is involved in a variety of physiological and pathological events [65]. However, the two forms of OPN complicate investigations of the biological functions of OPN (Figure 2).

### 6.1. sOPN

Under physiological conditions, sOPN is expressed in smooth muscle, bone, kidney, brain, mammary gland, and immune organs and is involved in wound healing, bone remodeling, and biomineralization [66,67]. DCs, lymphocytes, eosinophils, neutrophils, and other immune cells also express sOPN [68]. Immune cells produce sOPN to promote cell proliferation, adhesion, and migration as well as cell motility, fusion, and survival [69].

Under pathological conditions such as Th1- and Th17-related diseases, including viral infections, SLE, MS, IBD, and RA, sOPN modulates T-helper cell phenotypes by directly influencing macrophages, DCs, and T cells [70,71,72,73,74]. Through actions on macrophages, sOPN upregulates IL-12 production and enhances Th1 development via avb3 integrin binding and simultaneously downregulates IL-10 production through CD44 [70]. sOPN promotes IL-12 expression in DCs, which determines the Th1-polarizing capacity of DCs [71]. sOPN enhances CD3-mediated IFN-γ production in human T cells [72]. T-bet-mediated sOPN expression by T cells is decisive for CD4+ T cells polarized to be Th1 cells. Elevated levels of DC-derived OPN have been observed in encephalomyelitis and induce IL-17 expression in T cells [73]. DCs from MS patients produce increased levels of OPN to stimulate IL-17 and IFN-γ production in CD4+ T cells [74]. These data provide robust support for the role of sOPN in Th1- and Th17-polarized viral infections and autoimmune diseases.

### 6.2. iOPN

Physiologically, iOPN exhibits an important role in cell motility, cytoskeletal rearrangement, and mitosis. Compared to sOPN, less information has been obtained concerning the role of iOPN in various diseases. iOPN plays a role in viral infections and B cell development and function [45,75,76,77]. Viral antigens induce iOPN through TLRs in plasmacytoid dendritic cells (pDCs) and enhance IFN-α secretion, mediating the Th1 response, whereas the IFN-α produced by pDCs downmodulates iOPN in conventional dendritic cells (cDCs) and prevents an uncontrolled Th17 response [75,76]. The deletion of iOPN in NK cells impairs the expansion of these cells following IL-15 treatment, leading to a defective antiviral immune response [45]. The ICOS ligand on B cells activates ICOS on T cells, leading to the release of p85α and a complex formation with iOPN in T cells [45]. This complex translocates into the nucleus and binds Bcl-6, protecting it from proteasome-mediated degradation and thereby promoting Tfh cell differentiation [45]. Tfh cells release OPN, sustaining the B-cell response to antigens in germinal centers [45]. Furthermore, sOPN can promote immunoglobulins, particularly IgG3 and IgM antibody production, from B cells [77]. Therefore, iOPN and sOPN display complex interactions in the development and function of B cells in humoral immunity.

Earlier studies on the role of OPN were limited to Th1- and Th17-related viral and autoimmune processes [45,77]. The first indirect evidence regarding the effect of OPN in Th2-related diseases appeared in 2001 [78]. Researchers prepared radiolabeled cDNA from cultured peripheral blood mononuclear cells (PBMCs) from hyper-IgE syndrome patients and controls. The cells were stimulated for precisely 24 h with PHA (1 mg/mL) to probe an array of 375 immunologically relevant genes, and significantly reduced OPN levels in the hyper-IgE syndrome patients were found unexpectedly [78]. Later, recombinant OPN (rOPN) was found to downregulate IL-4 secretion in T cells, polarizing these cells toward a Th1 phenotype [79]. These data suggest that through its Th1 cytokine effects, OPN might play a central role in regulating overwhelming Th2 immune reactions, reminiscent of the mutual inhibition of Th1/Th2 during differentiation. In contact hypersensitivity, sOPN was confirmed to attract myeloid DCs to lymph nodes draining the skin [80]. However, sOPN inhibited pDC migration into lymph nodes upon encountering OVA [81]. Therefore, additional research is needed to delineate the role of OPN in the Th2 response in allergic diseases, considering that OPN affects different DC subsets and T cells that express high levels of OPN receptors but respond to OPN with opposite effects on the Th2 response [82,83,84]. In the past 20 years, evidence has supported an active role of OPN in Th2-related inflammation in allergic airway diseases, including CRSwNP, AR, and allergic asthma.

## 7. The Role and Regulation of OPN in CRSwNP

**Figure 3:**
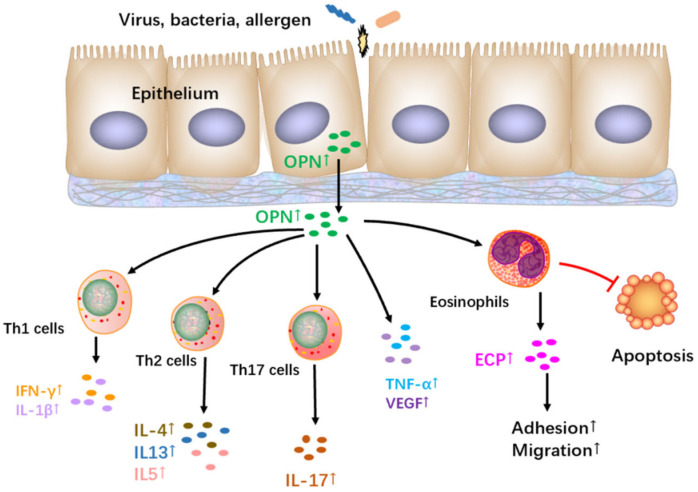
Epithelial-derived OPN is released in the setting of epithelial allergen or pathogenic challenge. OPN exerts an effect on diverse cells to stir up the production of IFN-γ, IL-1β, IL-4, IL-5, IL-13, TNF-α, ECP, and VEGF in the nasal mucosa, leading to the inflammation of CRSwNP and AR. In addition, OPN also promotes eosinophil adhesion, migration, and activation by enhancing ECP production but inhibits eosinophil apoptosis, enhancing the inflammation of CRSwNP and AR. OPN: osteopontin; VEGF: vascular endothelial growth factor; ECP: eosinophil cationic protein.

OPN has been identified by DNA microarray analysis as one of the 19 upregulated genes in polyp tissues [31]. OPN expression was higher in NP tissues than in controls based on immunohistochemical and qPCR analysis [31]. Positive immunohistochemical staining for OPN has been demonstrated in epithelial cells, infiltrating cells, submucosal glands, and the extracellular matrix. The main sources of OPN expression are the epithelial cells in polyp tissues [31,32]. NP tissues exhibit more OPN-positive cells than controls, and the number of OPN-positive cells is correlated with the number of tissue eosinophils. In vitro studies in a dispersed NP cell (DNPC) culture system revealed that OPN protein significantly promotes eosinophil migration and the production of eosinophil cationic protein (ECP) [31]. These findings substantiate the role of OPN in the eosinophilic inflammation in NP (Figure 3).

Our team discovered that the production of the Th1 signature cytokine IFN-γ, Th2 signature cytokines such as IL-4, IL-13, IL-5, and pro-inflammatory cytokines TNF-α and IL-1β is induced by rOPN in sinonasal mucosa explants [32]. This suggests that OPN might play a pivotal pro-inflammatory role during inflammation in the nasal mucosa, confirming that OPN might carry out a range of functions in NPs [32]. Recently, Du et al. demonstrated OPN-induced vascular endothelial growth factor (VEGF) expression by DNPCs through the phosphatidylinositol 3-kinase-protein kinase B (PI3K/AKT) and extracellular signal-regulated kinase 1/2 signaling pathways [85]. VEGF is significantly increased in NP tissues and might participate in the appearance of NPs and the substantial tissue edema occurring in NPs [85]. These results suggest that the OPN–VEGF axis promotes remodeling and edema in NPs (Figure 3).

As the epithelium constitutes the first line of defense against exogenous irritants, epithelial-cell-derived OPN regulation in various inflammatory environments was also investigated. As expected, a qPCR analysis by Liu et al. revealed that the TLR3 agonist Poly I:C significantly increased OPN mRNA production in polyp epithelial cells and normal nasal epithelial cells in vitro [31] and was the strongest stimulus for OPN mRNA expression. These results indicate that viral infections can induce OPN expression in NPs. Notably, cytokines such as IL-6, TNF-α, IL-17A, IFN-γ, and TGF-β can synergistically increase TLR3-mediated OPN production in epithelial cells ex vivo [31]. However, Liu et al. observed that IL-4 did not significantly affect OPN expression in epithelial cells [31]. In contrast, IL-4 significantly inhibited OPN expression in epithelial cells pre-conditioned by Poly I:C [31]. These data might indicate that IL-4 inhibits the viral response by regulating OPN in the nasal polyp immune network. Interestingly, we found that the expression of OPN decreased significantly following IL-4 treatment in cultured nasal mucosa [32]. This discrepancy might result from differences in the study models. We generated sinonasal mucosa explants, while Liu et al. utilized polyp epithelial cells. Furthermore, our data are in accordance with the fact that Th2 signature cytokines such as IL-4 inhibit OPN production in cDCs. Meanwhile, VEGF was found to induce OPN expression in DNPC, which could form a positive feedback loop between VEGF and OPN [85].

## 8. The Role and Regulation of OPN in AR

Limited information is available on the role of OPN in AR (Figure 3). We determined that OPN is significantly upregulated in the nasal mucosa of AR adults compared to controls [29]. In support of this observation, OPN expression was primarily expressed by epithelial cells and some inflammatory cells in the lamina propria [29]. In children with AR, increased serum and nasal OPN expression were positively correlated with eosinophilia and ECP levels [86]. Mechanistically, OPN can inhibit eosinophil apoptosis and promote eosinophil adhesion in vitro. Furthermore, OPN mediates eosinophil migration and activation through the PI3K pathway [87]. Recent studies have demonstrated that an elevated serum OPN is correlated with circulating IL-17 and Th2 cytokines [87,88]. A study utilizing house-dust-mite-stimulated PBMCs from children with AR revealed that OPN could enhance the expression of Th2 signature cytokines and increase Th17 responses [87,88]. However, these results contradict the evidence that OPN downregulates Th2 responses, as discussed in previous paragraphs. These discrepancies might be due to OPN’s influence on different cell types. This possibility is supported by the fact that OPN promotes Th2 effector responses by regulating pDCs when administered during the allergen-sensitization phase in an asthma model [82]. The precise mechanism by which OPN modulates the Th2 reaction in AR requires additional in-depth investigation.

Our team provided the earliest data regarding OPN regulation in AR. We found that Clara cell 10-kDa protein (CC10), an anti-inflammatory cytokine, markedly suppresses OPN mRNA expression in the local nasal mucosa in a CC10 knockout murine model of AR [29]. Our study further demonstrated that CC10 could regulate OPN expression in OVA-stimulated mononuclear cells isolated from spleens and decrease the OPN-induced production of Th2-related cytokines in a BEAS-2B cell line [29]. These studies suggest that CC10 might exert its inhibitory biological function by regulating OPN in AR. Later, O’Neil et al. found that natural exposure to pollen does not influence OPN expression in AR patients [89]. Also, treating AR patients locally with a potent nasal glucocorticoid did not alter mucosal OPN expression during natural exposure to pollen [89]. However, we should interpret these results cautiously because only the local iOPN of AR patients was assessed using immunochemistry in that study. It must be emphasized that although there were no differences in iOPN production, this might not be true for sOPN. It is necessary to test sOPN in nasal lavage fluid (NLF) to determine how natural pollen exposure or glucocorticoids influence its expression. Thus, the roles of iOPN and sOPN in AR require additional investigation.

With the high prevalence of allergic diseases and obesity among schoolchildren in the world, the research investigating the relationship between obesity and AR has received increased attention. Zeng et al. found higher OPN levels in the nasal turbinates of obese AR mice compared to nonobese or control mice, suggesting that obesity can regulate OPN expression [88]. They also reported that leptin, an adipose-derived, energy-regulating hormone, might interact with OPN to promote Th17 responses in AR [88], further supporting the role of obesity in OPN regulation. Immunotherapy has recently become a promising treatment for AR, and numerous potential treatment biomarkers have been studied. Similarly, the role of OPN has been investigated, and findings suggested that OPN expression decreased after one year of immunotherapy [90]. Furthermore, OPN expression is positively correlated with Th2-related cytokines and negatively correlated with TGF-β and IL-10 expression [90]. Thus, these findings suggest that OPN behaves more like a pro-inflammatory cytokine in AR and could be a valuable biomarker for AR immunotherapy. In contrast to this study, however, an increase in OPN and a decrease in bee-venom-specific IgE and IgG, especially IgG4, were confirmed after five to six years of bee-venom-specific immunotherapy [91]. These opposite results might be attributed to the specific environment of allergen contact in the period of immunotherapy or possibly reflect various mechanisms of immune tolerance induced by immunotherapy over time or even underlying differences in the ethnicity of the subjects. Similarly, increased IL-4 levels with unchanged Th1 and Th2 cell ratios after grass immunotherapy have been described, and IL-4 production and the Th2 population increased significantly after one year of bee venom immunotherapy, contradicting the commonly accepted Th2–Th1 switch described previously [92,93]. Therefore, the mechanisms of OPN in different immunotherapies require further study and exploration. Recently, our team found that mice treated with let-7a miRNA exhibited significantly enhanced OPN levels in the nasal mucosa compared to control mice, suggesting that let-7a stimulates AR development by modulating OPN expression [94]. However, this finding also needs further validation.

The two forms of OPN complicate investigations of the biological functions of OPN in AR, and the modulatory role of OPN in AR calls for more detailed investigation. Questions such as how OPN balances different cytokine patterns to influence allergic inflammation in AR could serve as a central focus for future studies.

## 9. The Role and Regulation of OPN in Allergic Asthma

Of the three allergic airway diseases, the earliest data on OPN came from allergic asthma. Asthma is caused by genetic and environmental factors [95]. OPN is located on human chromosome 4q13 (mouse chromosome 5), and single-nucleotide genetic polymorphisms of the gene have been confirmed to be associated with body infection, autoimmune diseases, asthma, and cancer susceptibility [96,97,98,99]. One study reported an association between OPN gene polymorphisms and asthma or allergies in a Japanese population [99]. Individuals in this Japanese population carry the C allele at position 5891 in exon 6, which is more common in patients with MS, a Th1-biased disease. This population exhibits significantly decreased total serum IgE levels compared with noncarriers [99]. MS patients show significantly reduced allergen sensitization, fewer allergy symptoms, and a reduced risk of asthma compared with individuals without MS [99]. These results indicate a potential relationship between the OPN gene and allergic disorders. However, it is not clear whether the same polymorphism also implies a risk of MS, which would decrease the risk for asthma. Therefore, additional study is needed to explore the relationship between OPN gene polymorphisms and asthma in different populations. Investigation of this relationship also should be extended to upper airway diseases.

Recent studies on OPN expression in airways have confirmed that OPN demonstrates a high expression in asthma patients [100,101]. OPN is mainly expressed in bronchial epithelial cells, followed by airway glandular endothelial cells and inflammatory cells, including macrophages, eosinophils, mast cells, and lymphocytes [100,101]. Clinical studies showed that OPN levels in bronchoalveolar lavage fluid (BALF) and sputum from asthmatic patients were significantly higher than in healthy controls [102]. Other studies found higher serum OPN levels in adult asthma patients than in healthy controls [103]. Akelma et al. also obtained consistent results in a study of serum OPN levels in asthmatic children older than five years [104]. Similarly, in a study of mouse models of allergic inflammation initiated by OVA treatment, the OPN levels increased in BALF and lung tissue [105]. These results firmly support the essential role of OPN in asthma. Delimpoura et al. showed that OPN levels in the sputum from several asthma patients were significantly higher than in patients with mild and moderate asthma and were associated with inflammatory mediators involved in airway inflammation and remodeling [106]. Meanwhile, the level of OPN in smoking patients is higher than in non-smoking patients, and smoking asthma patients typically exhibit more severe symptoms than non-smoking asthma patients [107]. Thus, the level of OPN is clearly correlated with asthma severity.

Airway remodeling is a prominent pathophysiological feature of asthma, mainly manifested as thickening of the basement membrane, collagen deposition, angiogenesis, and increased numbers of myofibroblasts [108,109]. IL-4, IL-5, IL-13, TNF-α, and IL-1β play critical roles in airway remodeling [108,109]. OPN contributes directly or indirectly to this pathology. The thickness of the airway basement membrane in human asthma patients was positively correlated with OPN expression levels [110]. In addition, the upregulation of OPN in lung tissue and BALF was related to collagen content and smooth muscle proliferation in an OVA-induced mouse model [111]. Kohan et al. and Simoes et al. reported that, compared with wild-type mice, OVA-induced OPN knockout asthma mice displayed reduced subcutaneous fibrosis, airway migration, inflammatory cell infiltration, TGF-β1 and VEGF production, collagen deposition, and smooth muscle actin expression [111,112,113]. In addition, the administration of rOPN in the remodeling stage when acute lung inflammation has receded and structural changes become the prominent features, increased the production of IL-13 and MMP9 in the lung and induced basement membrane thickening in a mouse model of chronic disease [113]. Furthermore, OPN induces lung fibroblasts to switch to a pro-fibrogenic myofibroblast phenotype [114]. In a previous study by Xanthou et al., rOPN exerted anti-inflammatory effects when administered in the acute state of the disease [114]. The different effects of rOPN are primarily attributed to the use of different disease models and the timing of the administration of rOPN (Table 1). As a matter of fact, the findings of the two studies complement each other, indicating the dual role of OPN in different stages of the disease. Initially, OPN inhibits Th2 inflammation and subsequently mediates the airway repair response, resulting in airway remodeling. Therefore, OPN contributes to airway remodeling and fibrosis in allergic asthma.

Allergic asthma involves Th2 cells and Th2 effectors, including eosinophils and mast cells [115,116]. Paradoxically, on one side, osteopontin inhibits Th2-related inflammation; on the other side, it promotes eosinophil accumulation, a key element for the TH2-mediated immune response [81]. sOPN promotes the chemotaxis of eosinophils via a4b1-integrin [117]. A blocking antibody, 2K1, consistently recognizes OPN’s integrin-binding domain and inhibits eosinophil migration in vitro [117]. These results confirm the ability of OPN to recruit eosinophils into asthmatic airways. OPN levels are correlated with eosinophil accumulation in BALF in asthmatic patients in vivo, supporting these observations [118]. Regarding the effect of OPN on mast cells in asthma, FceRI-aggregation-induced mast cell degranulation is enhanced by OPN through avb3 integrin ex vivo [119]. Furthermore, OPN promotes mast cell migration, which substantiates its effect on mast cells in allergic asthma [120,121].

Until now, contradictory results on the role of OPN in the Th2 response of allergic asthma have been observed (Table 1). Xanthou et al. found that OPN has dual effects in acute asthmatic mouse models [81]. The administration of an anti-OPN-specific antibody before the initial sensitization stage significantly reduced airway inflammation, suggesting that OPN plays a pro-inflammatory role at this stage [81]. However, treatment with an anti-OPN antibody before the OVA challenge revealed a magnified inflammatory response, suggesting that OPN played an anti-inflammatory role in this phase [81]. Therefore, it was proposed that OPN plays different roles in different stages of the OVA-induced inflammatory process in mice. Furthermore, the dual effect of OPN was attributed to different DC subpopulation recruitment [81]. The inhibition of OPN led to increased numbers of pDC in draining lymph nodes before sensitization. In contrast, a substantial increase in the recruitment of cDCs to lymph nodes draining the lungs was noted following anti-OPN antibody treatment at the challenge stage [81]. These data are consistent with previous investigations in which pDCs were confirmed to prevent Th2 responses [122]. However, treatment with rOPN during the sensitization stage inhibited OVA-specific IgE production in a chronic asthma mouse model [113]. It is challenging to reconcile these discrepancies, which may be due, in part, to the multi-functional properties of OPN in regulating cytokine production and inflammatory cell recruitment via the different functional domains in OPN. This was confirmed in a tolerance induction model where the SLAYGLR motif of sOPN enhanced the regulatory action exhibited in the tolerogenic context [123]. Whether similar effects can be demonstrated in human systems necessitates additional investigation. Furthermore, whether this holds true for upper airway diseases should be elucidated. The results obtained for upper airway diseases indicated that OPN has a pro-inflammatory effect in ex vivo explants, as discussed above [32], underscoring the urgent need to investigate OPN functions in animal models of CRSwNP and AR. In humans, asthma has been shown to be aggravated through multiple complex mechanisms and steps, ultimately leading to irreversible airway damage. Therefore, OPN exhibits different effects in the development of these allergic airway diseases that require additional investigation.

**Table 1:** Effects of osteopontin in different murine allergic asthma models.

Asthma Model	Modalities	Findings	References
An acute model	Anti-OPN antibodies at sensitization and challenge stage	Different DC subpopulation recruitment	[81]
An acute model	rOPN at challenge stage	Inhibited OVA-specific IgE production	[81]
A tolerance induction model	rOPN at sensitization stage	Induced accumulation of IFN-β-producing plasmacytoid dendritic cells and regulatory T cells in mediastinal lymph nodes	[123]
A chronic disease model	rOPN at challenge stage	Enhanced remodeling	[113]

rOPN—recombinant osteopontin; DC—dendritic cells.

Although OPN has been investigated in some detail in allergic asthma, information concerning the regulation of OPN in asthma remains scarce. Corticosteroids, the first-line treatment in asthma, similar to AR, also inhibit OPN production in mice with allergic asthma [124]. It is well-documented that smoking or exposure to secondhand smoke among asthmatics increases asthma-related morbidity and disease severity [125]. OPN levels are significantly higher in smoking asthmatics compared to non-smoking asthmatics, suggesting that smoking might contribute to disease severity in asthma by regulating OPN expression [126].

To elucidate whether aging or viral infections influence the pathology of asthma, six-week or twelve-week-old BALB/c mice were sensitized to OVA with or without poly(I:C) [127]. The twelve-week-old mice showed elevated levels of OPN in BALF and OPN mRNA expression in the lungs compared to the six-week-old mice [127]. Poly(I:C) induced remarkably elevated OPN levels in BALF and OPN mRNA expression [127]. Therefore, OPN expression in asthma is modulated by aging and viral infections. Whether other factors affect OPN production in asthma should be investigated further.

## 10. Clinical Relevance

Much has been learned concerning the role and regulation of OPN in disease pathology in allergic airway diseases over the past 20 years (Table 2). We now consider how to integrate this knowledge into clinical applications. Local or systemic OPN has been used as a diagnostic and prognostic biomarker in asthma (Table 3). However, whether we can use plasma or local OPN as a novel marker of disease severity and treatment efficacy for upper airway diseases requires additional detailed investigation.

The modulation of OPN may offer new treatment options for these allergic airway diseases, but additional research is required (Table 3). Researchers agree that neutralizing OPN effects can mediate the migration of eosinophils and mast cells, mast cell degranulation, and airway remodeling in vivo and in vitro [119,120,121]. However, the exploitation of rOPN or anti-OPN antibodies has pleiotropic effects on different immune cells and on a range of allergic reactions in vivo, and the data, such as the effect on Th2 response, have been contradictory [81,113]. Therefore, a more physiologically relevant mouse asthma model is urgently needed to evaluate the potential effect of the two methods. Furthermore, different anti-OPN antibodies might be exploited in different allergic airway diseases. These different antibodies could recognize different functional domains of the OPN molecule, resulting in various possible influences in the different disease processes.

## 11. Summary and Opinions

In this review article, we discussed the role of epithelial-cell-derived OPN in the pathogenesis of allergic airway diseases and summarized its clinical applications in those diseases. Data from human and animal models strongly support that epithelial-cell-derived OPN is a critical effector cytokine involved in Th2-biased airway inflammation. The disturbance of the highly controlled release of OPN may be an essential mechanism underlying CRSwNP, AR, and allergic asthma epithelial pathophysiology. As such, the research on the role of OPN in CRSwNP, AR, and asthma is still in its early stages. Several questions still need to be answered. 1. How do different OPN isoforms regulate eosinophilic inflammation by binding distinct OPN receptors in CRSwNP, AR, and asthma? 2. In CRSwNP, AR, and asthma, the detailed mechanisms of iOPN and sOPN-mediated functional activities need additional clarification. 3. The contradictory effects of epithelial-cell-derived OPN on Th2 responses require further investigation in CRSwNP, AR, and asthma. 4. OPN is a biomarker for the evaluation of disease treatment in clinical trials in cancers and autoimmune diseases. It must be elucidated whether OPN can be used as a biomarker for CRSwNP, AR, and asthma. 5. Although OPN can be used as a biomarker in immunotherapy, its precise function and mechanism require additional study. 6. Concerning the imbalanced investigations of OPN in upper and lower airway diseases, more attention should be afforded to CRSwNP and AR. In addition, OPN lacks preclinical investigation in upper airway diseases. Therefore, OPN urgently needs to be investigated as a target in mouse models of inflammatory upper airway disease.

## Figures and Tables

**Table 2:** The regulation of OPN in allergic airway diseases.

Regulatory Factors	Effect on OPN
IL-1β, TNF-α, IFN-γ, IL-6, IL-17A, IL-13, TGF-β	Upregulation
Leptin	
MicroRNA let-7a	
Smoking	
Aging	
Viral infection	
IL-4	Downregulation
Clara cell 10-kDa protein	
Corticosteroids	

OPN, osteopontin.

**Table 3:** Possible clinical relevance of OPN in current diseases.

OPN Manipulation	Function	Application
Local or systemic OPN	A diagnostic and prognostic biomarker	Asthma [103], cancers [37], hepatitis [37]
Anti-OPN antibodies	Promoting or inhibiting inflammation in different setting of diseases	Asthma [37], cancers [37], hepatitis [37], collagen-induced arthritis [26]
OPN siRNA	Inhibiting Th1-related inflammation	Cancers [37], hepatitis [37]
Recombinant OPN	Enhancing remodeling in airways and inhibiting Th1/Th17-related inflammation	Asthma [113], collagn-induced arthritis [22]
ASK8007	Blocking the function of OPN	Rheumatoid arthritis [20]

OPN, osteopontin; siRNA, small interfering RNA.
